# A Comparison of Changes in the Fatty Acid Profile of Human Milk of Spanish Lactating Women during the First Month of Lactation Using Gas Chromatography-Mass Spectrometry. A Comparison with Infant Formulas

**DOI:** 10.3390/nu11123055

**Published:** 2019-12-14

**Authors:** Silvia Sánchez-Hernández, Adelaida Esteban-Muñoz, Rafael Giménez-Martínez, María José Aguilar-Cordero, Beatriz Miralles-Buraglia, Manuel Olalla-Herrera

**Affiliations:** 1 Department of Nutrition and Bromatology, University of Granada, 18071 Granada, Spain; aidaem@ugr.es (A.E.-M.); rafaelg@ugr.es (R.G.-M.); olalla@ugr.es (M.O.-H.); 2 Programme in Nutrition and Food Science, University of Granada, 18071 Granada, Spain; 3 Instituto de Investigación Biosanitaria, 18071 Granada, Spain; 4 Department de Nursing, University of Granada, 18071 Granada, Spain; 5 Institute of Food Science Research (CIAL), CSIC-UAM, 28049 Madrid, Spain; beatriz.miralles@csic.es

**Keywords:** fatty acids, human milk, infant formula, GC-MS/MS, LC-PUFA

## Abstract

Breastfeeding is the ideal way to provide infants with the nutrients they need for healthy growth and development. Milk composition changes throughout lactation, and fat is one of the most variable nutrients in human milk. The aim of this study was to determine the main differences between the fatty acid (FA) profile of human milk samples (colostrum, transitional, and mature milk group) and infant formulas. Human milk samples were provided by lactating women from Granada. Moreover, different commercial infant formulas were analyzed. FAs were determined using gas chromatography coupled with mass spectrometry. According to the results, oleic acid was the predominant monounsaturated fatty acid (41.93% in human milk and 43.53% in infant formulas), while palmitic acid was the most representative saturated fatty acid (20.88% in human milk and 23.09% in infant formulas). Significant differences were found between human milk groups and infant formulas, mainly in long-chain polyunsaturated FAs (LC-PUFAs). The content of araquidonic acid (AA) and docoxahexaenoic acid (DHA) was higher in human milk (0.51% and 0.39%, respectively) than in infant formulas (0.31% and 0.22%, respectively). Linoleic acid (LA) percentage (15.31%) in infant formulas was similar to that found in human milk (14.6%). However, α-linolenic acid (ALA) values were also much higher in infant formulas than in human milk (1.64% and 0.42%, respectively).

## 1. Introduction

Breastfeeding is the ideal way to provide young children with the nutrients they need for healthy growth and development [1]. The World Health Organization (WHO) and the United Nations International Emergency Fund for Children (UNICEF) adopted measures in 2002 to promote global health. As a part of this, the global strategy for optimal food use in infants and young children recommended breastfeeding from the first hour of life, continuing with exclusive breastfeeding during the first 6 months of life and further breastfeeding up to 2 years of age which is supplemented with other foods [2,3]. Data published in 2016 by UNICEF indicates that, overall, only 43% (2 out of 5) of children continue to receive exclusive breastfeeding at 6 months of age [4]. This is because some mothers cannot or choose not to breastfeed and instead use infant formulas (IF) as a substitute. IF are manufactured foodstuffs for feeding newborns and babies that attempts to match, as much as possible, the composition of human milk (HM), especially the lipid profile [5]. Lipids are the largest source of energy in human milk. Triacylglycerols (TAGs) represent 98–99% of total fats and their properties are determined by the length of and degree of unsaturation of fatty acids (FAs) esterified to the glycerol backbone [6]. Milk composition changes throughout lactation, and fat is one of the most variable nutrients in human milk. Lipid content changes according to the stage of lactation and time of day, and during feeding. Whilst in human milk the composition of fatty acids is dynamic and modulated by maternal diet, infant formulas have a much less complex composition than human milk fat [6,7,8]. This can provide challenges in attempting to ensure normal or typical fatty acid intake in breastfed infants and in establishing fatty acid targets when developing infant formulas [8].

Human milk contains the parent essential fatty acids (EFA) linoleic acid (LA, C18:2 n-6) and α-linolenic acid (ALA, C18:3 n-3), and n-3/n-6 very long-chain polyunsaturated fatty acids (LC-PUFA) [9]. A balanced amount of these fatty acids is required for normal maturation and functioning of the nervous system [10]. These fatty acids are also associated with the development of allergic diseases and inflammatory responses [11]. They regulate growth, alongside visual, cognitive, and motor development during the first year of life [12,13,14,15].

LC-PUFA are present in human milk in concentrations greater than in other commercially available milks [16]. This is biologically relevant since the two major LC-PUFA are arachidonic acid (AA, C20:4 n-6) and docosahexaenoic acid (DHA, C22:6 n-3). These accumulate in the fetal retina and brain during the last trimester of pregnancy and the early period of postnatal development when milk represents the only source of fat [13,16].

Infant formulas provide all of these essential nutrients for adequate growth and development. These nutrients include the LA and ALA, as required by regulatory agencies [17], although the addition of LC-PUFA as AA and DHA is not mandated [18].

In recent years, variability of the fatty acid profile in human milk and in infant feeding has become very important. For this, the aim of the present study was: 

(1) To determine the main differences between the fatty acid profile of human milk samples at three stages of lactation (colostrum, transitional, and mature milk group) in women from Granada and compare the fatty acid profile of human milk with different commercially available infant formulas in Spain. This will provide new fatty acid data through use of novel analytical techniques. 

(2) To study the relationship between different milk samples according to their FA composition and determine which fatty acids are mostly responsible for the differences found between human milk samples and infant formulas.

## 2. Materials and Methods

### 2.1. Chemicals and Reagents

Fatty acid methyl esters (Supelco 35 Component FAME Mix) were obtained from Sigma-Aldrich SL (Madrid, Spain). N-hexane, isopropanol, anhydrous sodium sulfate, undecanoic acid (C11:0), methyl acetate, sodium methoxide, methanol, oxalic acid, and diethyl ether were acquired from Panreac Química SL (Panreac AppliChem, Barcelona, Spain) and Sigma-Aldrich (Merck, Munich, Germany). All reagents were of analytical grade.

### 2.2. Subject 

The present research was carried out in the obstetrics department of one of the six regional hospitals of Andalusia “Hospital Universitario Virgen de las Nieves”. Of all of the participants who formed part of this study, the three samples of human milk (colostrum, transitional, and mature milk) were donated by only thirteen lactating mothers due to the complexity of obtaining these samples. Details of the study were explained to all mothers who voluntarily gave written consent to participate. The characteristics of the study sample were as follows: women aged 18–40, who had given birth to healthy babies in the “Hospital Universitario Virgen de las Nieves”. This also constituted the inclusion criteria. 

Ethical approval for this study was provided by the “Hospital Virgen de las Nieves” Ethics and Scientific Committee and the trial was registered at ClinicalTrials.gov.

The mean age of participating mothers was 32 ± 4.7 years, of which eight reported this being their first lactation/child (N1, N2, N3, N5, N6, N10, N11, and N12).

The main characteristics of sampled mothers are shown in Table 1.

### 2.3. Milk Samples

Thirty-eight milk samples were categorized according to the length of time post-partum. Samples obtained between the 1st and 5th day post-delivery were assigned to the colostrum group (n = 13, 1.75 ± 0.53 days) with the Marmet manual extraction technique being used for delivery [19]; samples obtained between the 6th and 15th day post-delivery were assigned to the transitional group (n = 13, 12.83 ± 4.43 days); and samples obtained after the 15th day post-delivery were assigned to the mature milk group (n = 12, 24.75 ± 9.77 days). For both of the latter groups, milk extraction was achieved by means of a mechanical breast pump (Medela^®^, Medela, Switzerland) following the manufacturer’s instructions. Milk from each breast was obtained at both the beginning and end of each feed.

All human milk samples collected from study participants were aliquoted and immediately stored at −70 °C until extraction.

In addition to the human milk samples, seven different initiation formulas (0–6 months) for full-term infants were also analyzed. These are the most commonly consumed infant formulas and include brands such as Nestlé, Combiotik, Blemil, Nutribén, and Almirón, and were purchased in different commercial and agricultural areas of the market. They were randomly coded as IF 1–7 (Table S1). All samples were analyzed in triplicate.

### 2.4. Total Lipid Content and Fat Extraction

Infant formulas were reconstituted in water following the manufacturer’s instructions.

The human milk samples and infant formulas were extracted with a mixture of solvents according to the method of Hara and Radin [20].

First, 500 µL of milk was mixed with 1.8 mL of n-hexane, isopropanol (3:2, *v*/*v*), then and homogenized. Next, 1.2 mL of aqueous sodium sulfate was added and centrifuged to separate the layers. The organic layer was evaporated using nitrogen (N2). The aqueous phase was re-extracted, and the lipid recovered and stored at −18 °C in n-hexane: isopropanol (4:1, *v*/*v*) until the fatty acid methyl esters (FAME) were prepared.

### 2.5. Preparation of Methyl Esters for Gas Chromatographic Analysis

Fatty acid methyl esters (FAME) were obtained after following the base-catalyzed transesterification method described by Christie WW. [21]. The previously evaporated sample was dissolved in 0.5 mL of n-hexane. 40 μL of methyl acetate and 80 μL of sodium methoxide in methanol (0.5 M) were added. The solution was mixed for 30 s and left for 15 min at room temperature, at which time the reaction was stopped by adding 30 μL of a saturated solution of oxalic acid in diethyl ether. After a brief agitation, the mixture was centrifuged at 1500× g for 2 min and the supernatant, containing FAME, was collected into chromatography vials.

### 2.6. Chromatographic and Mass Operating Conditions

Determination of fatty acids was conducted using a mass spectrometer with tandem quadrupole model QUATTRO micro GC (WATERS, Milford, MA, USA), equipped with a split/splitless injector. A Flame Ionization Detector (FID) type detector with ionization mode EI+ at 300 °C was used, measuring a mass range of 45 to 450 and a 35 min chromatogram, and a capillary column with a length of 30.0 m and a diameter of 250 μm. The temperature of the initial oven was 100 °C with a maximum of 350 °C. The injection volume was 1 μL and the split ratio was set at 10.0 and split flow 10.0 mL/min. 

All data was collected using MassLynx V4.1 software (Waters Inc., 2010, Milford, MA, USA). Peaks were identified by comparing retention times with standard mixtures. Fatty acids were quantified by comparing the peak area of each compound with that of the standard.

### 2.7. Analytical Validation

Validation was carried out by studying the parameters of linearity, linear range, limit of detection (LOD), and limit of quantification (LOQ), following the guidelines for the validation of analytical methods (AOAC, 2012). Linearity in all fatty acids was achieved in their dynamic range of between 250–1000 ppm. A total of 35 fatty acids were identified and quantified in the different milk samples. 

This is a highly sensitive and precise analytical method based on mass spectrometry combined with high resolution separation methods.

### 2.8. Statistical Analyses

The homogeneity of variance was assessed using the Levene test and normality of data distribution of the samples was examined with the Shapiro–Wilk test. 

Results of individual FA content of the different human milk samples and infant formulas were analyzed using one-way ANOVA followed by the Tukey test in order to compare significant variations between means (*p* < 0.05). Moreover, in order to verify the capacity of the FA analysis as a tool for human milk characterization, a multivariate discriminant analysis was performed. Graphical representation of this analysis allows the similarity of samples to be assessed according to their FA composition.

The significance level was set at 5% (*p* < 0.05) in all tests. SPSS 15.0 for Windows (IBM SPSS Inc., Chicago, IL, USA) was used for data analyses.

In order to complete the examination of the fatty acid profile of human milk and infant formulas, the results obtained were subjected to discriminant analysis. This analysis is aimed at supporting the interpretation of complex multivariate data. It considers all observations as a single group with the aim of uncovering the variables with the greatest influence, so that observations can be grouped and predictive groups formed. The data obtained were expressed as means ± standard deviations (SD).

## 3. Results

### 3.1. Analytical Validation

**Figure 1:**
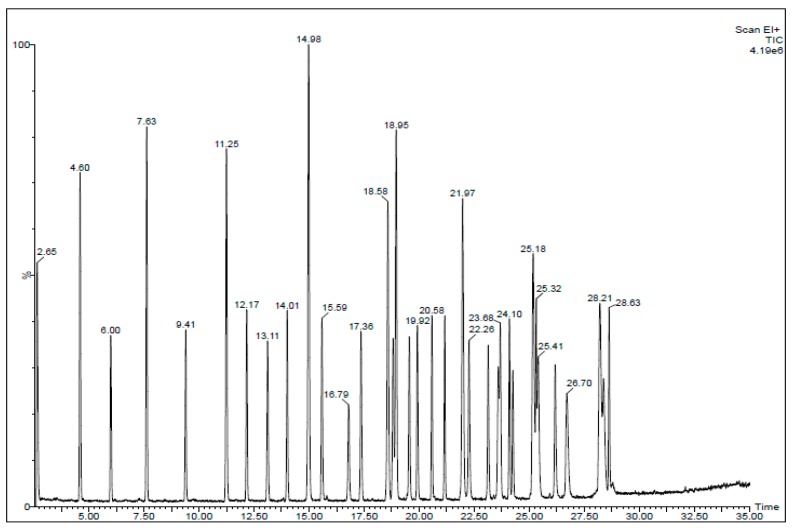
Chromatogram of a 35 component Fatty Acid Methyl Esters (FAME) mix of the standard.

A chromatogram of the standards of these fatty acids over a period of 35 min is shown in Figure 1.

Table 2 shows the linear range, retention time, adjusted linear equations, correlation coefficients, detection limits, and quantification limits of the standards.

A favorable correlation between the experimental data and the theoretical values was obtained with good linearity in the ranges evaluated and correlation coefficients (R2) greater than 0.998.

The highest LOD and LOQ corresponded to α-linolenic acid (C18:3 n-3) (1.8403 and 6.1344) and the lowest to arachidonic acid (C20:4 n-6) (0.1345 and 0.4482). These LOD and LOQ were compared with values previously reported by other authors for fatty acids using GC-MS/MS methods. 

### 3.2. Fatty Acid Profile

**Figure 2:**
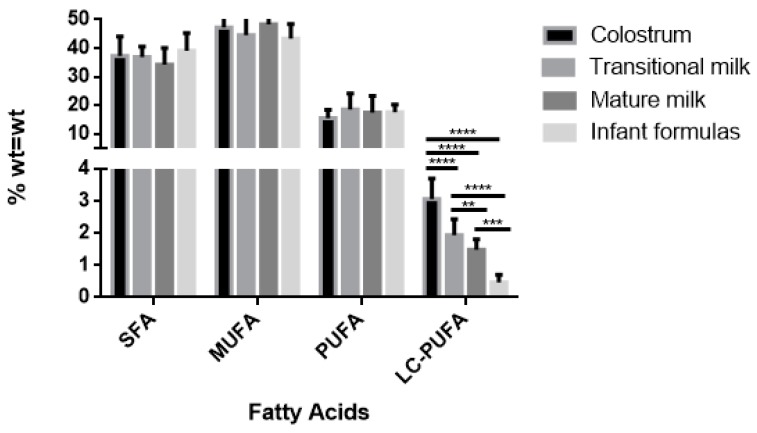
Fatty acid profile in human milk (colostrum n = 13; transitional milk n = 13; and mature milk n = 12) and infant formulas (n = 7). SFA: saturated fatty acids; MUFA: monounsaturated fatty acids; PUFA: polyunsaturated fatty acids and LC-PUFA: long-chain polyunsaturated fatty acids. Significant differences (** *p* < 0.01), (*** *p* < 0.001) and (**** *p* < 0.0001).

The fatty acid composition of colostrum, transitional, and mature milk samples of women from Granada, as well as that of infant formulas marketed are presented in Table 3, Table 4 and Table 5. Of the thirty-five standard fatty acids, a total of twenty-eight fatty acids were identified and quantified in our human milk and infant formula samples using GC-MS/MS analysis. These were grouped into saturated (SFAs), monounsaturated (MUFAs), and polyunsaturated (PUFAs) fatty acids. Figure 2 shows the distribution of the main fatty acid groups in the different samples of human milk and infant formulas.

#### 3.2.1. Saturated Fatty Acids (SFAs) 

The saturated fatty acids identified are shown in Table 3. 

The major SFA was C16:0, which is seen to decrease significantly when comparing the colostrum group to the transitional group (*p* < 0.05) and the mature group (*p* < 0.0001) within the milk samples (23.75%, 20.76% to 18.13% respectively). Nevertheless, a significant increase was observed between the colostrum group and the transitional and mature milk group (*p* < 0.0001) for C10:0 (0.18%; 1.06%, and 1.06%, respectively) and C12:0 (1.43%; 4.65%, and 3.82%, respectively).

Infant formulas showed significantly lower values of C18:0 than the colostrum (*p* < 0.0001), transitional (*p* < 0.01), and mature (*p* < 0.01) groups. In contrast, higher values of C12: 0 were found in infant formulas than the colostrum (*p* < 0.0001), transitional (*p* < 0.01), and mature (*p* < 0.001) groups. In addition, higher levels of C10: 0 were found relative to colostrum group (*p* < 0.0001), and higher C16: 0 relative to the transitional (*p* < 0.05) and mature (*p* < 0.001) groups. 

Thus, infant formulas have a higher proportion of SC-SFA (0.97% for colostrum, 1.51% for transitional milk, 1.70% for mature milk, and 2.01% for infant formulas) and MC-SFA (27.71% for colostrum, 30.38% for transitional milk; 26.05% for mature milk and 33.76% for infant formulas) than human milk. In contrast, they have a lower percentage of LC-SFA (6.41% for colostrum, 5.35% for transitional milk, 5.55% for mature milk, and 4.15% for infant formulas).

The other saturated fatty acids and overall SFA did not show significant differences between the milk groups.

#### 3.2.2. Monounsaturated Fatty Acids (MUFAs) 

The monounsaturated fatty acids identified are shown in Table 4.

The major MUFA was C18:1 n-9 Z, with similar values being found between human milk groups and infant formulas.

There was a decreasing trend between the colostrum group to the transitional and mature milk groups for C16:1 n-9 Z (1.52%, 1.23%, and 1.45%, respectively); C18:1 n-7 E (1.42%, 1.12%, and 1.11%, respectively); and C20:1 n-9 (0.97%, 0.52%, and 0.38%, respectively). 

In addition, significantly higher percentages were observed for these three fatty acids in human milk samples than in infant formulas (*p* < 0.01) (0.37% for C16:1 n-9 Z; 0.47% for C18:1 n-7 E; and 0.36% for C20:1 n-9). 

The other monounsaturated fatty acids and the total MUFA did not show significant differences between the milk groups and infant formulas.

#### 3.2.3. Polyunsaturated Fatty Acids (PUFAs) 

The polyunsaturated fatty acids identified are shown in Table 5.

The major PUFA was C18:2 n-6 Z, (LA), with similar values being found between human milk groups and infant formulas. In contrast, C18:3 n-3 (ALA) showed significantly higher values in infant formula than in the different human milk groups (*p* < 0.0001). Thus, the LA/ALA ratio showed statistically significant differences between the human milk group and infant formulas (*p* < 0.001).

C20:2 and C20:3 n-6 showed a significant decrease between the colostrum group and the transitional (*p* < 0.01) and mature (*p* < 0.0001) milk groups. Whilst measurements for C22:6 n-3 (DHA) did not show statistically significant differences, instead presenting similar trends between groups. 

**Figure 3:**
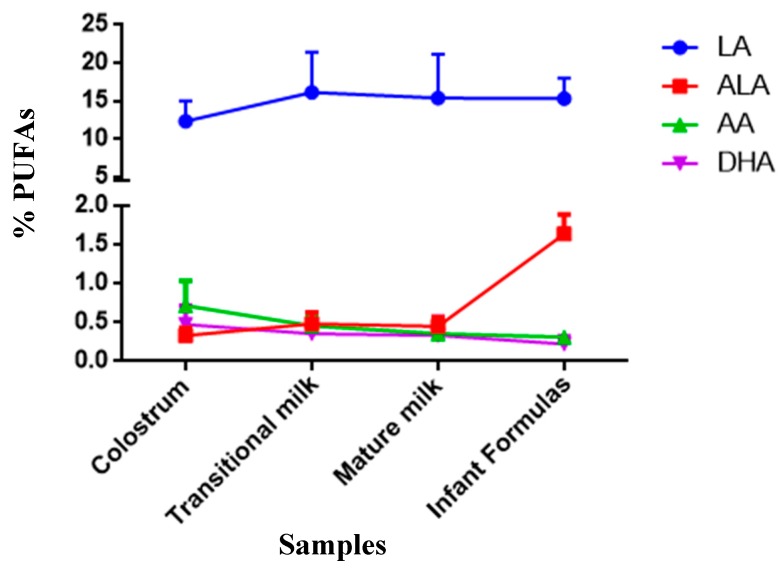
Trend of the majority of polyunsaturated fatty acids (PUFAs) in human milk (colostrum, transitional, and mature milk) and infant formulas. LA: linoleic acid; ALA: α-linolenic acid; AA: araquidonic acid; DHA: docoxahexaenoic acid.

With regards to LC-PUFA (C20–24), greater variability was seen between the human milk and infant formula groups. Figure 3 shows the variability of the main PUFAs in human milk and infant formulas. The latter group did not contain C20:2, GLA, and DGLA, whilst the percentages of AA and DHA were significantly lower than that found in human milk. 

The remaining polyunsaturated fatty acids and overall PUFA did not show significant differences between the milk groups and infant formulas.

### 3.3. Discriminant Analysis

Forty-five cases were used to develop a model that discriminates between the human milk and infant formula samples. Thirty-six predictor variables were introduced. Table 6 displays the discriminant functions obtained in the present analysis. 

In this case, our first discriminant function has a relative variance percentage close to 100% (87.58%), while the second and third discriminant function is only able to explain 11.03% and 1.39% of the variance of the data, respectively. The *p*-value of discriminant functions 1 and 2 are less than 0.05, whereby both discriminant functions are statistically significant with a 95.0% confidence level.

In addition, the canonical correlation of the first and second functions is closer to 1. These functions allowed 91.1% of groups to be classified (100% for infant formulas versus 83.3% of mature human milk). Finally, Wilk’s Lambda is also closer to 0 in the first two discriminant functions.

Taken together, results indicate that our first two discriminant functions are capable of separating the data much better than the third function.

**Figure 4:**
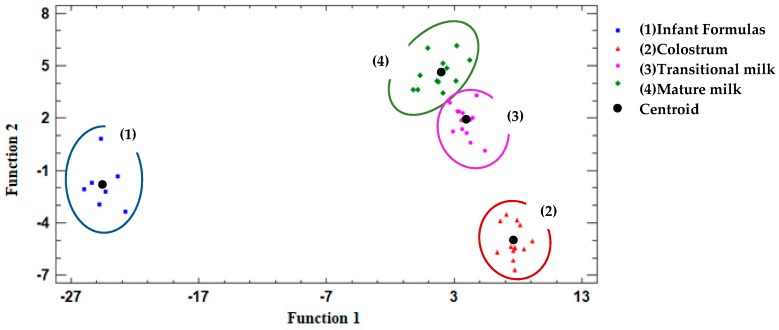
Representation of the different groups of milks studied (colostrum, transitional milk, mature milk, and infant formulas) based on the two significant functions according to the discriminant analysis performed. Centroid: average value of the discriminant function for each one of the samples.

A graphical representation of the two dimensions is shown in Figure 4, according to the first and second discriminating functions.

## 4. Discussion

The results of the present study show that the average characteristics relating to the fatty acid composition of human milk can be summarized as follows. Palmitic acid (≈20%), oleic acid (≈44%), and linoleic acid (≈15%) were the predominant fatty acids in the colostrum, transitional, and mature milk groups, and in the infant formulas. These represented between 76.67% and 81.94% of the total fatty acids. This trend is similar to that found by Yang et al., which was between 77.98% and 78.57% [22]. Next, we consider the results relating to myristic acid (≈4%) and stearic acid (≈6%).

All of these values were very similar to those described by other authors. Yang et al. [23] found similar values in palmitic acid (21.77%), myristic acid (3.91%), and stearic acid (5.14%); but found lower values in oleic acid (33.32%) and higher values in linoleic acid (22.54%). However, Zou et al. [24] reported more similar values with respect to palmitic acid (20.3%), linoleic acid (17.1%), myristic acid (4.9%), and stearic acid (6.0%). On the other hand, they also identified lower percentages of oleic acid (34.9%).

### 4.1. Saturated Fatty Acids (SFAs) 

Total saturated fatty acid (SFA) content remained stable across all of the milk groups [24]. Human milk typically contains approximately 34% to 47% saturated fatty acids, mainly palmitic acid (17–25%) [7,22]. Similar values were found in other studies [10,13,25]. The colostrum and infant formula groups presented similar percentages of this SFA (23.75% and 23.09%, respectively). However, in human milk, palmitic acid is esterified with triglycerides in position 2 (position β), whereas unmodified milk fat of infant formulas is esterified in positions 1 and 3. The specific distribution of fatty acids in the triglyceride plays a key role in the digestion and absorption of lipids. It appears that this modification of fat decreases the stability and quantity of calcareous soaps in feces, thus decreasing its consistency [24,25,26]. 

Other fatty acids, such as C10:0 and C12:0 increased from one lactation stage to the next. This trend was very similar to that found in another Spanish study [10] (0.66%, 1.66%, and 1.63%) for C10:0 and (3.49%, 6.97%, and 6.28%) for C12:0 in colostrum, transitional, and mature milk, respectively.

Therefore, infant formulas have a higher proportion of SC-SFA and MC-SFA than human milk, possibly due to the addition of vegetable oils [26]. MC-SFA are commonly supplemented and incorporated into these infant formulas because they can be directly absorbed by the portal vein and rapidly generate energy for infants [27]. However, this has been related to an increase in the level of total and low-density lipoprotein (LDL) cholesterol concentration in plasma leading to a high risk of cardiovascular disease. On the other hand, LC-SFA is reported to be neutral concerning its effects on lipoprotein cholesterol levels [28].

Despite this, overall lauric and myristic acid content is recommended to not exceed 20% of total fat in infant formulas, with content being 10.88% in the present case [29].

### 4.2. Monounsaturated Fatty Acids (MUFAs) 

Total monounsaturated fatty acid (MUFA) remained stable in all milk samples (45.99% for colostrum, 43.34% for transitional, and 46.99% for mature milk group). This trend has also been described by other authors [30]. Barreito et al. reported a percentage of 44.1%.

Oleic acid (C18:1 n-9) constitutes more than 90% of the total MUFAs, finding similar values in human milk groups and infant formulas. This is fundamentally linked to the consumption of olive oil, representing levels greater than 40% [6,15].

These values are higher than those found in other studies, both Spanish and European studies, possibly because southern Spain shows higher levels of adherence to the Mediterranean diet [31]. This may be due to the potentially high adherence to the Mediterranean diet of our participants which is composed of foods rich in oleic acid, especially for the high consumption of extra virgin oil. According to the data offered by ministry of agriculture, food and environment (MAGRAMA) in 2018, Andalusía purchases and consumes more extra virgin olive oil than any other autonomous community, accounting for 25.99% of the volume distributed across Spain (L) [32]. 

C16:1 n-9, C18:1 n-7, and C20:1 n-9 were the three most abundant MUFAs in human milk after oleic acid, with a percentage of around 3–4%, whilst the percentage present in infant formulas was 1.2%.

The results of a previous study indicate that the majority of commercially available IF do not contain scientifically recommended amounts of vaccenic acid, and that their fatty acid composition is deficient in comparison with human milk [33]. Vaccenic acid is the major trans-fatty acid in ruminant milk fat. It is unique in that it may provide cis-9, trans-11-octodecadienoic (cis 9, trans 11-C18:2; also known as rumenic acid) to the consumer through endogenous desaturation by the Δ-9 desaturase enzyme [34]. Rumenic acid is the most common conjugated linoleic acid (CLA) isomer and it has been shown to promote various beneficial health-related effects, including anti-carcinogenic, anti-atherosgenic, anti-diabetic, and immune-modulating effects, in addition to effects on body composition and fat metabolism [35,36,37].

### 4.3. Polyunsaturated Fatty Acids (PUFAs)

Concentrations of PUFA in human milk are relatively stable during the first year of life. AA typically constitutes 1% (0.72%) in colostrum and 0.5% in mature milk (0.36%); DHA is approximately equivalent to 0.5% in colostrum (0.47%) and 0.25% in mature milk (0.33%) [16]. Also, it typically contains approximately 12% to 26% n-6 PUFA and 0.8% to 3.6% n-3 PUFA [7].

Moreover, essential fatty acid (EFA) content varies depending on the stage of lactation, particularly LA levels (12.32% for colostrum; 16.10% for transitional; and 15.38% for mature milk). Zou et al. showed this trend in the results of their study (21.01%, 21.05%, and 25.58% for colostrum, transitional, and mature milk, respectively) [23], although the values of our study were lower. Values, however, were similar to those reported by Ribeiro et al. [38] (15.46% for samples 7 days postpartum and 16.20% for samples at 4 weeks postpartum).

DHA, AA, and DGLA content also showed a decrease according to the stage of lactation [38,39], finding higher values in colostrum than in transitional and mature milk groups [23]. Only a slight increase in GLA was observed, correlated with a reduction in inflammation after childbirth. GLA is the substrate for DGLA synthesis, another anti-inflammatory fatty acid [36,37,40] Fu et al. evaluated the DHA and arachidonic acid (AA) levels in human milk according to country and region, reporting similar values (0.42% for DHA and 0.71% for AA) for Spain to those found in the present study [41].

However, higher levels of ALA were observed in mature milk compared to the colostrum and transitional groups, as has also been described by other authors [15].

In infant formulas, LA percentage (15.31%) was similar to that found in human milk (14.6%). However, the content of AA (0.31%) and DHA (0.22%) was lower than in human milk (0.51% and 0.39%, respectively), especially when considering the colostrum stage. Conversely, ALA values were also much higher in infant formulas than in human milk (1.64% and 0.42%, respectively). DHA experiences a longer and more complicated synthesis which limits the conversion rate of ALA/DHA [42]. This trend is shown in Figure 2.

For this reason, infant formulas present a significantly higher percentage of n-3 PUFA than that seen in the different groups of human milk. This is due to their high amount of α-linolenic acid. 

Codex Alimentarius stipulate that the AA and DHA content of infant formulas should, at least, have the same concentration [43]. The content of these fatty acids in our infant formulas was 0.31% and 0.22%, respectively. These values being similar to those described by Chen et al. [27], which reported values of 0.41% for AA and 0.23% for DHA. 

These values are below estimated averages for AA and DHA in human milk samples studied by Brenna et al. [42]. These authors included 84 studies and reported that the worldwide mean concentration in human milk was 0.47% ± 0.13% for AA and 0.32% ± 0.22% for DHA. These values are very similar to those uncovered in the present sample (0.51% for AA and 0.39% for DHA).

The AA/DHA ratio did not vary with increasing milk maturation, however the LA/ALA ratio was significantly higher in the colostrum, transitional, and mature milk groups than in infant formulas (38.15%, 32.03%, 31.30%, and 9.53%, respectively). It is important to note that this ratio is within the guideline range of 5:1 and 15:1 suggested by the ESPHGAN Committee on Nutrition (European Society for Pediatric Gastroenterology Hepatology and Nutrition) [29].

### 4.4. Discriminant Analysis

An eigenvalue in discriminant analysis is the characteristic root of each function, that is, it is an indication of how well that function differentiates the groups, and the larger the eigenvalue is, the better the function differentiates the groups [44].

In the present study, the infant formula group shows left-handside displacement on the graph, while the human milk groups are found to the right of the graph. Differences between the colostrum, transitional, and mature human milk groups can be established. 

This indicates that all of the present samples can be grouped and differentiated from each other according to their fatty acid profile. Infant formulas are distinct from the human milk groups, largely due to the differences described in their LC-PUFAs. In addition, it is interesting to observe that the different human milk samples can be assigned to independent groups, following the observation that samples belonging to the colostrum group have a different composition than the other samples (transitional and mature groups).

In conclusion, this method was proven to be a useful tool for studying the relationships between oils according to their fat composition [44].

### 4.5. Limitations and Strenghts

Some limitations should be acknowledged. The small sample size used in the present study is a limitation, although we did not observe significant differences in the fatty acid profile of lactating women according to the anthropometric variables studied, such as weight, height, or BMI (normal BMI and those with a BMI above 25 kg/m^2^); this also being the case for the other variables described. 

On the other hand, data on human milk has been previously reported for many European countries and cultures but no recent data about milk from lactating women relates to southern Spain [10,22]. This means there is an interest in carrying out this study with lactating women in Granada. It also constitutes a strength of the present study that it provides new fatty acid data in human milk, as well as infant formulas that are currently commercial, through the use of novel analytical techniques (GC-MS/MS).

## 5. Conclusions

The outcome of the present study showed that the fatty acid profile of human milk samples varies throughout lactation. This variability in the profile of fatty acids between the different samples of human milk justifies that is not a static fluid and changes over time, adapting to the nutritional needs of the infant. However, infant formulas are rather uniform with respect to their composition.

According to the results, despite the fatty acid profile being similar in infant formulas and in human milk in terms of total SFA, MUFA, and PUFA; significant differences were found in some important fatty acids (such as ALA, GLA, DHA, or AA) between different human milk groups and infant formulas. Further, more evident statistically significant differences were observed between the colostrum group and the other samples.

Nevertheless, although infant formulas are enriched with the main LC-PUFAs, typically rich in LA, and in some cases with sufficient contributions of ALA, they tend to have low DHA, AA, and GLA content with respect to human milk. These fatty acids are important for fetal growth, and brain and retina development during pregnancy and the early years of life. Scientific evidence has shown that non-breastfed children suffer from a greater prevalence, severity, and longevity of diseases, not only during the time of breastfeeding but many years later.

Furthermore, the distribution of palmitic acid in infant formulas should resemble that of human milk as the specific distribution of fatty acids in triglyceride plays a key role in the digestion and absorption of lipids.

In conclusion, this experimental work can be used to ensure that infant formulas are as similar as possible to human milk and reproduces as close as possible the complexity of human milk composition. This is important in circumstances where breastfeeding is impossible, insufficient, or undesired.

## Figures and Tables

**Table 1:** Characteristics of the sampled mothers.

	Mean	SD	Minimum	Maximum
Age (years)	32	4.7	26	40
Height (cm)	163.6	7.00	150.0	172.0
Weight (kg)	69.6	13.50	47.4	90.8
BMI (kg/cm^2^)	26.2	5.33	18.4	33.4
Birth weight (kg)	2.9	0.79	1.2	3.8
Parity	1.6	0.92	1	3

BMI: body mass index; SD: standard deviation.

**Table 2:** Quality parameters for the chromatographic determination of fatty acid standards.

Fatty Acids	Linear Range (ppm)	t_r_ ± SD	Linear Equation	*R*	*R* ^2^	LOD (ppm)	LOQ (ppm)
Caprylic acid (C8:0)	250–1000	2.647 ± 0.006	y = 271.7940x + 813.2345	0.9999	0.9997	0.3362	1.1208
Capric acid (C10:0)	250–1000	4.597 ± 0.006	y = 362.0136x - 8269.8440	0.9999	0.9997	0.3223	1.0743
Undecanoic acid (C11:0)	250–1000	6.000 ± 0.000	y = 183.0014x - 4785.7425	0.9999	0.9998	0.4375	1.4583
Lauric acid (C12:0)	250–1000	7.623 ± 0.006	y = 403.2163x - 11475.9145	0.9998	0.9996	0.2929	0.9765
Tridecanoic acid (C13:0)	250–1000	9.400 ± 0.010	y = 197.6763x - 6117.3745	0.9999	0.9999	0.5236	1.7452
Myristic acid (C14:0)	250–1000	11.247 ± 0.006	y = 414,5987x - 8987,9605	0.9994	0.9988	0.1620	0.5400
Myristoleic acid (C14:1)	250–1000	12.167 ± 0.006	y = 217,1785x - 9140,8480	0.9993	0.9987	0.1514	0.5046
Pentadecanoic acid (C15:0)	250–1000	13.117 ± 0.012	y = 211,0244x - 6724,5895	0.9996	0.9993	0.2058	0.6862
Cis-10-pentadecenoic acid (C15:1)	250–1000	14.010 ± 0.010	y =221,6763 x - 9651,3250	0.9999	0.9998	0.2058	0.6862
Palmitic acid (C16:0)	250–1000	14.980 ± 0.010	y = 663,2947x - 24193,3435	0.9999	0.9998	0.3793	1.2642
Palmitoleic acid (C16:1 n-9 Z)	250–1000	15.587 ± 0.006	y = 227,0054x - 8233,3830	0.9996	0.9991	0.1901	0.6337
Margaric acid (C17:0)	250–1000	16.793 ± 0.006	y = 147,9136x - 5182,7135	1.0000	1.0000	0.9664	3.2212
Cis-10-heptadecenoic acid (C17:1)	250–1000	17.357 ± 0.006	y = 229,9246x - 10188,1020	0.9999	0.9998	0.4456	1.4855
Stearic acid (C18:0)	250–1000	18.577 ± 0.006	y = 464,1352x - 13723,7815	0.9997	0.9995	0.2437	0.8123
Elaidic acid (C18:1 n-9 E)	250–1000	18.817 ± 0.006	y = 158.1385x - 2278.2970	1.0000	0.9999	0.6563	2.1877
Oleic acid (C18:1 n-9 Z)	250–1000	18.953 ± 0.006	y = 425.2101x - 7612.0155	0.9997	0.9993	0.2137	0.7122
Linolelaidic acid (C18:2 n-6 E)	250–1000	19.553 ± 0.006	y = 230.1367x - 15194.7850	0.9997	0.9994	0.2214	0.7380
Linoleic acid (C18:2 n-6 Z)	250–1000	19.920 ± 0.000	y = 223.0317x - 11160.5665	1.0000	0.9999	0.6918	2.3060
γ-linolenic acid (C18:3 n-6, GLA)	250–1000	20.580 ± 0.000	y = 212.2357x - 12443.1135	1.0000	0.9999	0.7184	2.3946
α-linolenic acid (C18:3 n-3, ALA)	250–1000	21.160 ± 0.000	y = 202.2944x - 9492.2895	1.0000	1.0000	1.8403	6.1344
Arachidic acid (C20:0)	250–1000	21.973 ± 0.015	y = 494.0689x - 16256.6330	0.9991	0.9983	0.1345	0.4482
Cis-11-eicosenoic acid (C20:1)	250–1000	22.263 ± 0.006	y = 241.1562x - 12764.6795	1.0000	1.0000	0.8160	2.7199
Cis-11,14-eicosadienoic acid (C20:2)	250–1000	23.130 ± 0.010	y = 211.9830x - 9814.0900	0.9998	0.9996	0.2733	0.9110
Heneicosanoic acid (C21:0)	250–1000	23.590 ± 0.010	y = 150.6156x - 2443.3555	1.0000	1.0000	1.6576	5.5254
Cis-8,11,14-eicosatrienoic acid (C20:3 n-6, DGLA)	250–1000	23.687 ± 0.006	y = 197.8801x - 7653.1285	0.9998	0.9996	0.2765	0.9218
Arachidonic acid (C20:4 n-6, AA)	250–1000	24.103 ± 0.006	y = 194.4791x - 7437.3125	0.9998	0.9995	0.2568	0.8560
Cis-11,14,17-eicosatrienoic acid (C20:3)	250–1000	24.253 ± 0.006	y = 141.3401x - 3637.9235	0.9992	0.9984	0.1398	0.4659
Behenic acid (C22:0)	250–1000	25.180 ± 0.010	y = 408.6182x - 10400.1250	1.0000	0.9999	0.7288	2.4292
Erucic acid (C22:1 n-9)	250–1000	25.313 ± 0.006	y = 221.7645x - 17072.2935	1.0000	0.9999	0.7788	2.5959
5,8,11,14,17-eicosapentadienoic acid (C20:5 n-3, EPA)	250–1000	25.407 ± 0.006	y = 199.8515x - 10168.5900	0.9997	0.9994	0.2339	0.7797
Cis-13,16-docosadienoic acid (C22:2)	250–1000	26.173 ± 0.006	y = 213.4314x - 12761.5080	1.0000	0.9999	0.6514	2.1713
Tricosanoic acid (C23:0)	250–1000	26.710 ± 0.010	y = 228.7863x - 7187.5190	0.9997	0.9994	0.2318	0.7728
Lignoceric acid (C24:0)	250–1000	28.213 ± 0.006	y = 443.1121x - 25091.8985	0.9999	0.9999	0.5220	1.7401
Nervonic acid (C24:1)	250–1000	28.373 ± 0.006	y = 228.3534x - 11611.2500	1.0000	1.0000	1.0994	3.6648
Cis-4,7,10,13,16,19-docosahexadienoic acid (C22:6 n-3, DHA)	250–1000	28.630 ± 0.000	y = 215.8342x - 19113.3950	1.0000	0.9999	0.5976	1.9921

t_r_: retention time; SD: standard deviation; *R*: correlation coefficient; *R*^2^: squared correlation coefficient; LOD: limit of detection; LOQ: limit of quantification.

**Table 3:** Saturated fatty acid (SFAs) composition of colostrum, transitional, and mature milks, and infant formulas (% wt = wt).

Fatty Acids (FAs)	Colostrum	Transitional Milk	Mature Milk	Infant Formula
Caprylic acid (C8:0)	0.72 ± 0.634	0.51 ± 0.130	0.64 ± 0.197	0.77 ± 0.271
Capric acid (C10:0)	0.18 ^a,b,d^ ± 0.160	1.06 ± 0.268	1.06 ± 0.173	1.04 ± 0.424
Lauric acid (C12:0)	1.43 ^a,b,d^ ± 0.768	4.65 ^e^ ± 1.578	3.82 ^f^ ± 1.158	7.45 ± 2.378
Tridecanoic acid (C13:0)	0.00	0.01 ± 0.020	0.00	0.01 ± 0.010
Myristic acid (C14:0)	3.79 ± 1.165	4.56 ± 1.420	3.68 ± 1.326	3.43 ± 1.517
Pentadecanoic acid (C15:0)	0.15 ± 0.140	0.22 ± 0.009	0.18 ± 0.093	0.15 ± 0.282
Palmitic acid (C16:0)	23.75 ^a,b^ ± 2.032	20.76 ^c^ ± 2.031	18.13 ^f^ ± 2.332	23.09 ± 2.669
Margaric acid (C17:0)	0.09 ± 0.133	0.07 ± 0.104	0.08 ± 0.109	0.05 ± 0.109
Stearic acid (C18:0)	6.23 ^d^ ± 1.192	5.54 ^e^ ± 0.906	5.46 ^f^ ± 0.781	3.81 ± 0.516
Arachidic acid (C20:0)	0.04 ^d^ ± 0.096	0.02 ^e^ ± 0.074	0.03 ^f^ ± 0.075	0.33 ± 0.110
Lignoceric acid (C24:0)	0.04 ± 0.144	0.00	0.00	0.00
SFA	36.05 ± 5.266	37.49 ± 3.153	33.19 ± 4.899	37.31 ± 4.522
SC-SFA (C8-C10)	0.97 ^a,b,d^ ± 0.622	1.51 ± 0.346	1.70 ± 0.266	2.01 ± 0.465
MC-SFA (C12-C16)	27.71 ^d^ ± 3.305	30.38 ^c^ ± 2.856	26.05 ^f^ ± 4.370	33.76 ± 4.219
LC-SFA (> C17)	6.41 ^b,d^ ± 1.153	5.35 ± 0.901	5.55 ^f^ ± 0.735	4.15 ± 0.451

SD: standard deviation; SFA: saturated FAs; SC-SFA: short-chain SFA; MC-SFA: medium-chain SFA; LC-SFA: long-chain SFA. (a) Significant differences (*p* < 0.05) between colostrum and transitional milk groups, (b) significant differences (*p* < 0.05) between colostrum and mature milk groups, (c) significant differences (*p* < 0.05) between transitional and mature milk groups, (d) significant differences (*p* < 0.05) between colostrum and infant formula groups, (e) significant differences (*p* < 0.05) between transitional and infant formula groups, (f) significant differences (*p* < 0.05) between mature and infant formula groups.

**Table 4:** Monounsaturated fatty acid (MUFAs) composition of colostrum, transitional, mature milk, and infant formulas (% wt = wt).

Fatty Acids (FAs)	Colostrum	Transitional Milk	Mature Milk	Infant Formula
Myristoleic acid (C14:1)	0.02 ^a^ ± 0.064	0.08 ± 0.085	0.06 ± 0.0796	0.18 ± 0.328
Cis-10-pentadecenoic acid (C15:1)	0.00	0.00	0.00	0.01 ± 0.034
Palmitoleic acid (C16:1 n-9 Z)	1.52 ^d^ ± 0.320	1.23 ^e^ ± 0.667	1.45 ^f^ ± 0.416	0.37 ± 0.159
Vaccenic acid (C18:1 n-7 E)	1.42 ^a,b,d^ ± 0.148	1.12 ^e^ ± 0.161	1.11 ^f^ ± 0.099	0.47 ± 0.103
Oleic acid (C18:1 n-9 Z)	42.11 ± 5.228	39.81 ± 4.608	43.88 ± 7.041	43.54 ± 3.428
Gadoleic acid (C20:1 n-9)	0.97 ^a,b,d^ ± 0.253	0.52 ^e^ ± 0.173	0.38 ± 0.097	0.36 ± 0.046
Erucic acid (C22:1 n-9)	0.00	0.03 ± 0.066	0.01 ± 0.035	0.00
MUFA	45.99 ± 5.750	43.34 ± 4.754	46.99 ± 6.796	44.86 ± 3.689

SD: standard deviation; MUFA: monounsaturated fatty acids. (a) Significant differences (*p* < 0.05) between colostrum and transitional milk groups, (b) significant differences (*p* < 0.05) between colostrum and mature milk groups, (c) significant differences (*p* < 0.05) between transitional and mature milk groups, (d) significant differences (*p* < 0.05) between colostrum and infant formula groups, (e) significant differences (*p* < 0.05) between transitional and infant formula groups, (f) significant differences (*p* < 0.05) between mature and infant formula groups.

**Table 5:** Polyunsaturated fatty acid (PUFAs) composition of colostrum, transitional, and mature milk, and infant formulas (% wt = wt).

Fatty Acids (FAs)	Colostrum	Transitional Milk	Mature Milk	Infant Formula
Cis-9,12-hexadecadienoic (C16:2 n-4)	0.00	0.00	0.01 ± 0.032	0.00
Linolelaidic acid (C18:2 n-6 E)	0.01 ± 0.047	0.00	0.00	0.03 ± 0.072
Linoleic acid (C18:2 n-6 Z (LA))	12.32 ± 2.643	16.10 ± 5.325	15.38 ± 5.754	15.31 ± 2.667
γ-linolenic acid (C18:3 n-6 (GLA))	0.00 ^a,b^	0.04 ^e^ ± 0.064	0.07 ^f^ ± 0.087	0.00
α-linolenic acid (C18:3 n-3 (ALA))	0.33 ^d^ ± 0.103	0.48 ^e^ ± 0.149	0.45 ^f^ ± 0.121	1.64 ± 0.247
Cis 11,14-eicosadienoic acid (C20:2)	0.98 ^a,b,d^ ± 0.282	0.64 ^e^ ± 0.332	0.40 ^f^ ± 0.084	0.00
Dihono-γ-linolenic acid (C20:3 n-6 (DGLA))	0.74 ^a,b,d^ ± 0.274	0.49 ^e^ ± 0.139	0.34 ^f^ ± 0.098	0.00
Arachidonic acid (C20:4 n-6 (AA))	0.72 ^a,b,d^ ± 0.321	0.46 ± 0.114	0.36 ± 0.073	0.31 ± 0.078
Cis 13,16-docosadienoic acid (C22:2)	0.03 ± 0.084	0.00	0.00	0.00
Cis 4,7,10,13,16,19-docosahexadienoic acid (C22:6 n-3 (DHA))	0.47 ± 0.240	0.36 ± 0.140	0.33 ± 0.240	0.22 ± 0.099
PUFA	15.94 ± 2.504	17.80 ± 5.038	17.48 ± 5.828	17.55 ± 2.667
UFA	62.68 ± 6.83	63.07 ± 3.62	65.78 ± 5.88	60.94 ± 6.22
SFA/UFA	0.62 ± 0,20	0.59 ± 0,09	0.53 ± 0,14	0.64 ± 0.17
n-3 PUFA	0.77 ^d^ ± 0.308	0.84 ^e^ ± 0.165	0.79 ^f^ ± 0.350	1.81 ± 0.232
n-6 PUFA	14.11 ^a^ ± 2.333	16.38 ± 4.979	16.14 ± 5.907	14.83 ± 1.756
LC-PUFA (C20-C24)	3.06 ^a,b,d^ ± 0.641	1.85 ^c,e^ ± 0.381	1.43 ^f^ ± 0.260	0.54 ± 0.158
LA/ALA	38.15 ^d^ ± 17.980	32.03 ^e^ ± 10.030	31.30 ^f^ ± 15.370	9.53 ± 0.667
AA/DHA	1.74 ± 0.812	1.54 ± 0.683	1.43 ± 1.016	1.33 ± 0.361

SD: standard deviation; PUFA: polyunsaturated fatty acids; UFA: unsaturated FAs; SFA/UFA: saturated FAs / unsaturated FAs; LC-PUFA: long-chain PUFA; LA/ALA: linoleic acid/α-linolenic acid; AA/DHA: araquidonic acid/docoxahexaenoic acid. (a) Significant differences (*p* < 0.05) between colostrum and transitional milk groups, (b) significant differences (*p* < 0.05) between colostrum and mature milk groups, (c) significant differences (*p* < 0.05) between transitional and mature milk groups, (d) significant differences (*p* < 0.05) between colostrum and infant formula groups, (e) significant differences (*p* < 0.05) between transitional and infant formula groups, (f) Significant differences (*p* < 0.05) between mature and infant formula groups.

**Table 6:** Discriminant functions that predict the types of milks analyzed based on fatty acid levels.

Discriminant Functions	Eigenvalue	Relative Percentage (%)	Canonical Correlation	Lambda De Wilks	Chi-Squared	*p*-Value
1	126.3990	87.58	0.9961	0.0002	210.5987	0.0000 *
2	15.9167	11.03	0.9610	0.0197	94.2629	0.0282 *
3	2.0021	1.39	0.8166	0.3331	26.3837	0.8214

* : Significant differences (*p* < 0.05).

## Supplementary Materials

The following are available online at https://www.mdpi.com/2072-6643/11/12/3055/s1, Table S1: Basic formulation details of Infant Formulas (IF).
