# Supplementary material for: A Comparison of Changes in the Fatty Acid Profile of Human Milk of Spanish Lactating Women during the First Month of Lactation Using Gas Chromatography-Mass Spectrometry. A Comparison with Infant Formulas
Source: Nutrients. 2019 Dec 14;11(12):3055. doi: 10.3390/nu11123055 (PMC6950188; doi:10.3390/nu11123055)
Supplement: Supplementary file 1 [file nutrients-11-03055-s001.pdf]

Supplementary materials

Table S1. Basic formulation details of Infant Formulas (IF).

	Fat Total	SFA	MUFA	PUFA	Palmitic Acid	LA (w-6)	ALA (w-3)	AA (w-6)	DHA (w-3)	Added ingredients
	g/100mL					mg/100mL				
Infant Formula 1	3.4	1.5	-	-	-	388	48	13.1	13.1	w-3 y w-6 LC_PUFA, FOS, Nucleotidos
Infant Formula 2	3.5	1.3	-	-	-	454	52	6.9	6.9	GOS, Nucletidos, DHA
Infant Formula 3	3.4	1.5	-	-	-	400	80	10	10	GOS/FOS (9:1), DHA/AA, Nucleotidos
Infant Formula 4	3.5	1.2	1.6	0.7		600	70	12	7	GOS, PCL (w-6 y w-3)
Infant Formula 5	3.5	1.5	-	-	0.8	418	46	7	7	w-3 y w-6 AGPI-CL, FOS, Nucleotidos
Infant Formula 6	3.6	1.4	-	-	-	573	69	-	-	Nucleotidos
Infant Formula 7	3.5	1.3	-	-	-	429	52	7.4	6.7	GOS, Nucleotidos, DHA

SFA: saturated fatty acids; MUFA: monounsaturated fatty acids; PUFA: polyunsaturated fatty acids; LA: LA: linoleic acid; ALA: α-linolenic acid; AA: arachidonic acid; DHA: docoxahexaenoic acid; LC-PUFA: long-chain polyunsaturated fatty acids; FOS: frutooligosaccharides; GOS: galactooligosaccharides.
